# Supplementary material for: The Systems Biology Research Tool: evolvable open-source software
Source: BMC Syst Biol. 2008 Jun 29;2:55. doi: 10.1186/1752-0509-2-55 (PMC2446383; doi:10.1186/1752-0509-2-55)
Supplement: Additional file 1 — SBRT Archive. An archive of the current version of the Systems Biology Research Tool. [file 1752-0509-2-55-S1.zip › sbrt-1.4.0/doc/developers_guide/api/sbrt/shell/text/class-use/PipeListFormat.html]

Uses of Class sbrt.shell.text.PipeListFormat


|  |  |  |
| --- | --- | --- |
| **Overview** \| **Package** \| **Class** \| **Use** \| **Tree** \| **Deprecated** \| **Index** \| **Help** | |  |
| PREV   NEXT | **FRAMES**    **NO FRAMES**     **All Classes** |  |


---


## **Uses of Class sbrt.shell.text.PipeListFormat**

| Packages that use PipeListFormat | |
| --- | --- |
| **sbrt.shell.text** | Provides classes and interfaces for defining formats for basic objects. |
| **sbrt.shell.text.fba** | Provides classes and interfaces for defining formats for objects relevant to Flux Balance Analysis. |

Uses of PipeListFormat in sbrt.shell.text

| Methods in sbrt.shell.text that return PipeListFormat | |
| --- | --- |
| `static PipeListFormat<java.lang.String>` | `PipeListFormat.getBasic()`             Returns a `PipeListFormat` that does not format or parse the elements supplied to it. |
| `PipeListFormat<V>` | `EqualsMapFormatV1.getListFormat()`             Returns the format used for lists of values. |
| `PipeListFormat<java.lang.String>` | `AppInputFileLineFormatVersion1.getListFormat()`             Returns the format used for lists of values. |
| `PipeListFormat<V>` | `ColonMapFormatV1.getListFormat()`             Returns the format used for lists of values. |

Uses of PipeListFormat in sbrt.shell.text.fba

| Methods in sbrt.shell.text.fba that return PipeListFormat | |
| --- | --- |
| `PipeListFormat<java.util.Set<LinearComb<java.lang.String>>>` | `FluxCapFormatV1.getListFormat()`             Returns the format used for lists of values. |
| `PipeListFormat<Interval>` | `ConstraintsFileLineFormatV1.getListFormat()`             Returns the format used for lists of values. |
| `PipeListFormat<java.lang.Double>` | `FluxVectorFormatV1.getListFormat()`             Returns the format used for lists of values. |
| `PipeListFormat<ChemRxn>` | `IrfFormatV1.getListFormat()`             Returns the format used for lists of reactions. |
| `PipeListFormat<Interval>` | `ConstraintsFileLineFormatV2.getListFormat()`             Returns the format used for lists of values. |
| `PipeListFormat<LinearComb<java.lang.String>>` | `FluxomeSolFileLineFormatV1.getListFormat()`             Returns the format used for lists of values. |

---


|  |  |  |
| --- | --- | --- |
| **Overview** \| **Package** \| **Class** \| **Use** \| **Tree** \| **Deprecated** \| **Index** \| **Help** | |  |
| PREV   NEXT | **FRAMES**    **NO FRAMES**     **All Classes** |  |


---
